# Mégaœsophage: un cas historique

**DOI:** 10.11604/pamj.2018.29.192.4708

**Published:** 2018-04-02

**Authors:** Houda Meyiz, Mounia Elyousfi

**Affiliations:** 1 University of Sidi Mohammed Ben Abdellah, Faculty of Medicine and Pharmacy, Department of Gastroenterology C, Fez, Morocco

**Keywords:** Mégaœsophage, achalasie, fibroscopie, sphincter inferieur de l´œsophage, Megaoesophagus, achalasia, fibroscopy, lower oesophageal sphincter

## Abstract

Achalasia, also known as cardiospasm or megaoesophagus is a rare disorder of unknown origin. It is a primary motor disorder of the esophagus characterized by the absence of the esophageal peristalsis and by an incomplete or an absent relaxation of the lower oesophageal sphincter. Common symptoms include dysphagia, regurgitations and retrosternal pain. At an advanced level, fibroscopy can show a dilation of the lower esophagus and a shrinkage that can be easily passed through with a lifting at the level of the cardia. Oesogastroduodenal transit shows esophageal dilation and allows to estimate esophageal evacuation speed. Esophageal manometry is the gold standard diagnostic tool because it can show the absence of esophageal body peristalsis, the elevation of lower esophageal sphincter pressure and the absence of complete relaxation of the lower esophageal sphincter during swallowing. The different therapeutic strategies aim to reduce lower oesophageal sphincter pressure. We report the case of a patient with immense extension of the oesophagus with typical achalasia appearance. The patient aged 33 years had capricious low dysphagia evolving since childhood associated with regurgitations and retrosternal pain within a context of a 10kg weight loss. Clinical examination was unremarkable. Oesophagogastroduodenoscopy (OGD) was performed showing very dilated atonic oesophagus with food stasis and very tight cardia easily passed through with a lifting. EGD-transit showed immense dilation of the oesophagus without oesogastric junction abnormality suggesting achalasia. Surgical treatment was recommended.

## Image en médecine

L'achalasie, également appelée cardio-spasme ou mégaœsophage est une affection rare de cause inconnue. C'est un trouble moteur primitif de l'œsophage caractérisé par l'absence du péristaltisme œsophagien et par une relaxation incomplète ou absente du sphincter inferieur de l'œsophage. Les symptômes habituels associent une dysphagie, des régurgitations et des douleurs rétro-sternales. La fibroscopie permet de montrer, dans les formes plus évoluées, une dilatation du bas œsophage et un rétrécissement aisément franchi avec un ressaut au passage du cardia. Le transit œsogastroduodénal met en évidence une dilatation de l'œsophage et permet d'estimer la vitesse de vidange du contenu œsophagien. La manométrie œsophagienne est le principal outil diagnostic en mettant en évidence l'absence de péristaltisme du corps de l'œsophage, l'élévation de la pression du sphincter inférieur de l'œsophage et l'absence de relaxation complète de ce dernier à la déglutition. Les différentes stratégies thérapeutiques ont pour but la réduction de la pression du SIO. Nous rapportons une observation d'un patient présentant une dilatation monstrueuse de l'œsophage illustrant un aspect typique d'une achalasie. Il s'agit d'un patient âgé de 33 ans, avec notion de dysphagie basse, capricieuse, évoluant depuis l'enfance, associée à des régurgitations avec douleurs rétro-sternales, évoluant dans un contexte d'amaigrissement chiffré à 10kg. L'examen clinique et sans particularité. Une FOGD a été réalisée objectivant un œsophage très dilaté atone, siège d'une stase alimentaire avec un cardia très serré franchi avec ressaut. Le TOGD a montré une dilatation monstrueuse de l'œsophage sans anomalie de la jonction œsogastrique évoquant une achalasie. Un traitement chirurgical a été proposé.

**Figure 1:**
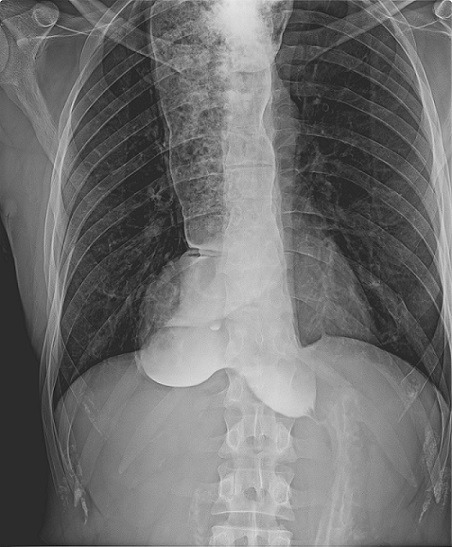
Clichés de TOGD objectivant la dilatation œsophagienne

